# Adults with blood cancers respond to booster, not initial dose of COVID-19 vaccine

**Published:** 2022-08

**JULY 7, 2022** - People with hematologic malignancies—or blood cancers including leukemia, lymphoma, and multiple myeloma—have an impaired immune system due to their disease and its treatment, putting them at risk of severe COVID-19 infection and experiencing a reduced response to COVID-19 vaccination. In a recent study published by Wiley online in CANCER, a peer-reviewed journal of the American Cancer Society, less than half of patients with hematologic malignancies mounted detectable antibodies after initial COVID-19 vaccination, but 56% of “nonresponders” produced antibodies after receiving a booster dose.

For the study, Thomas Ollila, MD, of Brown University, and his colleagues retrospectively analyzed antibody responses to initial and booster COVID-19 vaccination in 378 patients with hematologic malignancies.

Anti-SARS-CoV-2 antibodies were detected in the blood of 181 patients (48%) after initial vaccination with one of three U.S. Food and Drug Administration (FDA)-authorized or approved COVID-19 vaccines, and patients with active cancer or those recently treated with an immune cell–depleting therapy were least likely to produce these antibodies. Among patients who did not mount an antibody response following initial vaccination, responses were observed after a booster dose in 48 of 85 (56%) patients who were assessed.

By the end of February 2022, 33 patients (8.8%) developed a COVID-19 infection, with three COVID-19-related deaths (0.8%). Although there was no significant link between post-vaccination antibody response and incidence of COVID-19 infection, no patient with antibody responses died from COVID-19.

Also, no patient who received tixagevimab plus cilgavimab was diagnosed with a COVID-19 infection. Tixagevimab and cilgavimab are antibody therapies that bind to non-overlapping portions of the SARS-CoV-2 spike protein, preventing the virus from binding to and infecting cells. The FDA authorized the combination therapy for emergency use during the COVID-19 pandemic as a way to help prevent COVID-19 infection in certain individuals.

“Our findings build on the wealth of literature showing that patients with hematologic malignancies have an impaired response to COVID vaccination. Importantly, we show that many of these patients who did not respond initially will in fact have a response to booster vaccination,” said Dr. Ollila. “Moreover, when we looked at outcomes, we found that deaths from COVID-19 in the patient population we reviewed only occurred in those with undetectable antibodies, and nobody who received prophylactic antibody therapy was diagnosed with COVID-19. This suggests to us the importance of checking antibody levels in these patients and arranging prophylactic antibody therapy.”

Dr. Ollila encourages providing booster vaccines for patients and prioritizing prophylactic antibody therapy when indicated. “This is real world evidence that these actions can save lives,” he said.

***Link to Study: http://doi.wiley.com/10.1002/cncr.34354***

***Full citation:** “Seroconversion and outcomes after initial and booster COVID-19 vaccination in adults with hematologic malignancies.” Thomas A. Ollila, Rebecca H. Masel, John L. Reagan, Shaolei Lu, Ralph D. Rogers, Kimberly J. Paiva, Rashida Taher, Ella Burguera-Couce, Adam S. Zayac, Inna Yakirevich, Rabin Niroula, Peter Barth, and Adam J. Olszewski. CANCER; Published Online: July 11, 2022 (DOI: 10.1002/cncr.34354)*.

